# Feasibility and efficacy of minimally invasive limited resection for primary duodenal gastrointestinal stromal tumors: a retrospective cohort study

**DOI:** 10.1186/s12893-024-02417-z

**Published:** 2024-04-27

**Authors:** Longhang Wu, Miao Liu, Xianchao Lin, Congfei Wang, Yuanyuan Yang, Haizong Fang, Heguang Huang, Ronggui Lin, Fengchun Lu

**Affiliations:** 1 Department of General Surgery, Fujian Medical University Union Hospital, Fuzhou, Fujian China; 2 Fujian Clinical Research Center for Digestive System Tumors and Upper Gastrointestinal Diseases, Fuzhou, Fujian China; 3 Department of Gastrointestinal Endoscopy Nursing, Fujian Medical University Union Hospital, Fuzhou, Fujian China

**Keywords:** Duodenal tumor, Gastrointestinal stromal tumor, Duodenectomy, Laparoscopic, Robotic

## Abstract

**Background:**

The primary duodenal gastrointestinal stromal tumor (GIST) is a rare type of gastrointestinal tract tumor. Limited resection (LR) has been increasingly performed for duodenal GIST. However, only a few studies reported minimally invasive limited resection (MI-LR) for primary duodenal GIST.

**Methods:**

The clinical data of 33 patients with primary duodenal GIST from December 2014 to February 2024 were retrospectively analyzed including 23 who received MI-LR and 10 who received laparoscopic or robotic pancreaticoduodenectomy (LPD/RPD).

**Results:**

A total of 33 patients with primary duodenal GIST were enrolled and retrospectively reviewed. Patients received MI-LR exhibited less OT (280 vs. 388.5min, *P*=0.004), EBL (100 vs. 450ml, *P*<0.001), and lower morbidity of postoperative complications (52.2% vs. 100%, *P*=0.013) than LPD/RPD. Patients received LPD/RPD burdened more aggressive tumors with larger size (*P*=0.047), higher classification (*P*<0.001), and more mitotic count/50 HPF(*P*=0.005) compared with patients received MI-LR. The oncological outcomes were similar in MI-LR group and LPD/RPD group.

All the patients underwent MI-LR with no conversion, including 12 cases of LLR and 11 cases of RLR. All of the clinicopathological data of the patients were similar in both groups. The median OT was 280(210-480) min and 257(180-450) min, and the median EBL was 100(20-1000) mL and 100(20-200) mL in the LLR and the RLR group separately. The postoperative complications mainly included DGE (LLR 4 cases, 33.4% and RLR 4 cases, 36.4%), intestinal fistula (LLR 2 cases, 16.7%, and RLR 0 case), gastrointestinal hemorrhage (LLR 0 case and RLR 1 case, 9.1%), and intra-abdominal infection (LLR 3 cases, 25.0% and RLR 1 case, 9.1%). The median postoperative length of hospitalization was 19.5(7-46) days in the LLR group and 19(9-38) days in the RLR group. No anastomotic stenosis, local recurrence or distant metastasis was observed during the follow-up period in the two groups.

**Conclusions:**

Minimally invasive limited resection is an optional treatment for primary duodenal GIST with satisfactory short-term and long-term oncological outcomes.

## Introduction

Gastrointestinal stromal tumors (GISTs) are the most common mesenchymal tumors of the gastrointestinal tract, of which 60% are located at the stomach and 30% are located at the small intestines [1]. The primary duodenal gastrointestinal stromal tumor is a rare type of GIST, which accounts for approximately 4-5% of all GISTs. The most common location of duodenal GIST is the second (D2) portion of the duodenum, compared to the first (D1), the third (D3), and the fourth (D4) portion of the duodenum [2].

Complete surgical resection is the mainstay of curative treatment for primary non-metastatic duodenal GISTs [3, 4]. Various procedures have been advocated for resectable duodenal GISTs, including limited resection (LR) and extended resection [5]. Extended resection mainly indicates pancreaticoduodenectomy (PD), while LR includes wedge resection, segmental duodenectomy or pancreas-sparing total duodenectomy. A recent European multicenter cohort study demonstrated that limited resection has similar oncological outcomes and a lower incidence of morbidity than PD for duodenal GIST [6]. However, the oncological outcomes of minimally invasive limited resection (MI-LR) for duodenal GISTs have not been investigated even after the minimally invasive approaches have been gradually adopted for duodenal surgery. MI-LR which includes laparoscopic limited resection (LLR) and robotic limited resection (RLR) are being increasingly performed in many centers with the advantage of preservation of organ function and rapid recovery. However, the experience and evidence regarding such minimally invasive therapeutic strategy is limited, especially the oncological outcomes, of which many are case reports [7].

In the present study, we performed MI-LR for 23 patients with primary duodenal GIST with satisfactory short-term and long-term oncological outcomes compared with minimally invasive pancreaticoduodenectomy.

## Methods

### Study design and patients

Patients diagnosed as duodenal GIST who underwent MI-LR, LPD or RPD at Fujian Medical University Union Hospital from December 2014 to February 2024 were considered eligible and included. Lesions originated from non-duodenal legions or metastatic duodenal GIST were excluded. Several preoperative examinations were performed for a complete evaluation of the duodenal tumor, including endoscopy, serum tumor markers, upper gastrointestinal contrast, ultrasonography, contrast-enhanced computed tomography (CT), and magnetic resonance imaging (MRI). Endoscopic ultrasonography (EUS) was adopted if necessary. Surgical approaches like MI-LR, LPD or RPD were determined by multidisciplinary assessment. Neoadjuvant imatinib was not been administered considering its benefit of improving the rate of R0 resection or overall survival has not been proven [8].

The clinicopathological data of the patients were collected and prospectively recorded in a computer database. The characteristics of the data included age, gender, clinical presentation, tumor characteristics, surgical details, postoperative complication, length of stay (LOS), and follow-up details. The tumor characteristics included tumor location (D1, D2, D3, D4), tumor size, pathology (confirmed with histological and immunohistochemical analysis), mitotic count, CD117, CD34, Ki67, and risk classification. The risk classification was determined according to the consensus guidelines of the National Institutes of Health (NIH), based on tumor location, tumor size, and mitotic count [9]. The surgical details included resection status, procedures, operative time (OT), and estimated blood loss (EBL).

### Surgical approaches

The patient was placed in a supine position with two legs separated. For LLR, A 12-mm optical port was positioned below the umbilicus. A 5-mm and a 12-mm ports were placed at the right upper abdomen for the surgeon. Two 5-mm ports were placed at the left upper abdomen for the first assistant. The trocar placement of RLR was similar to that of LLR, as previously described [10]. For RLR, a total intra-abdominal exploration to exclude metastasis was adopted before docking of the robotic operative system.

**Fig. 1:**
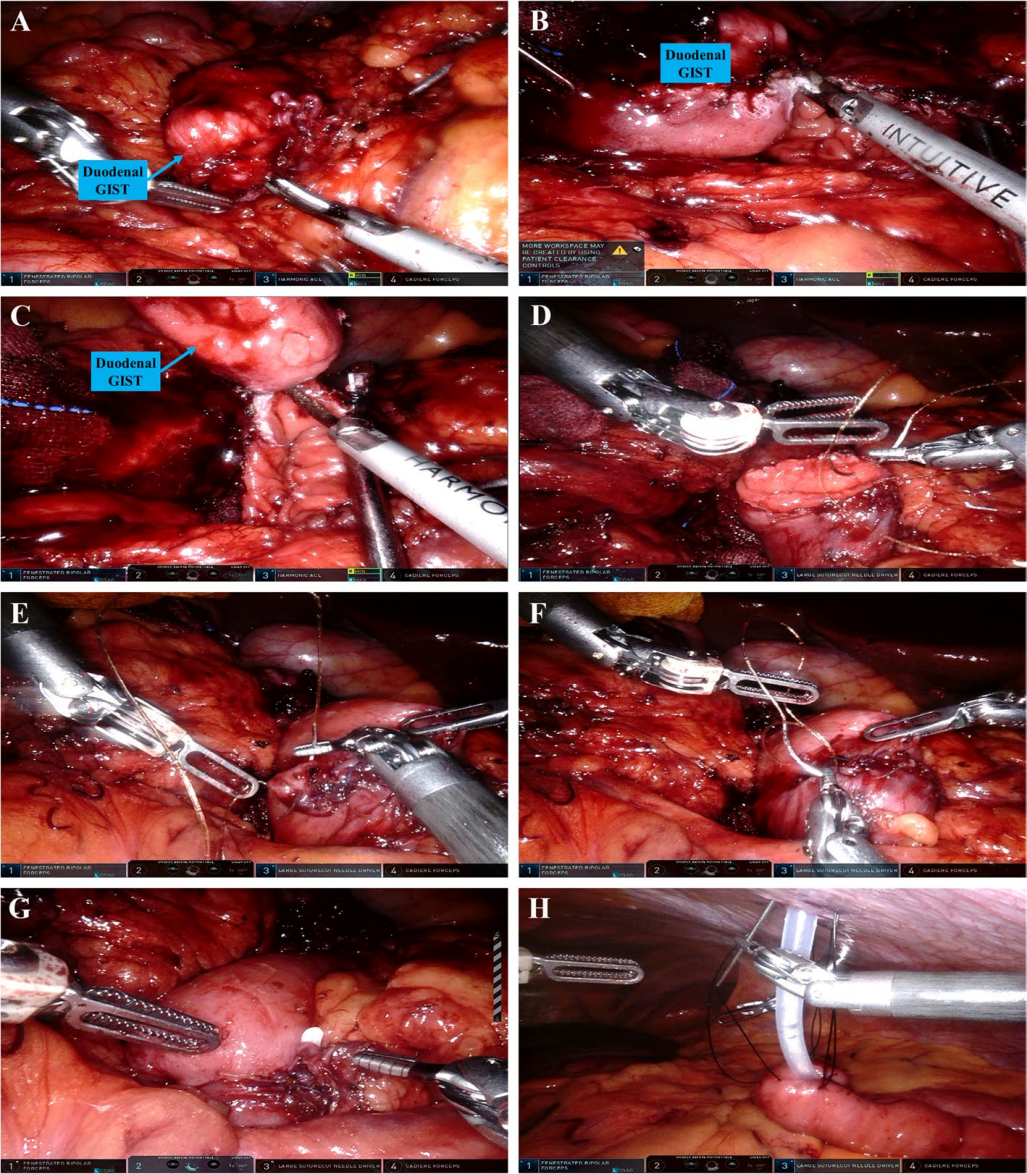
Robotic wedge resection for a GIST at the second portion of the duodenum. **A**. Exploration of the duodenal GIST; **B**-**C**. Wedge resection of the duodenal GIST; **D**-**E**. Closure of duodenal defect with continuous suture; **F**. Suture of the seromuscular layer of the duodenum for enhancement; **G**. View after the closure of duodenal defect; **H**. Nutritional jejunostomy

**Fig. 2:**
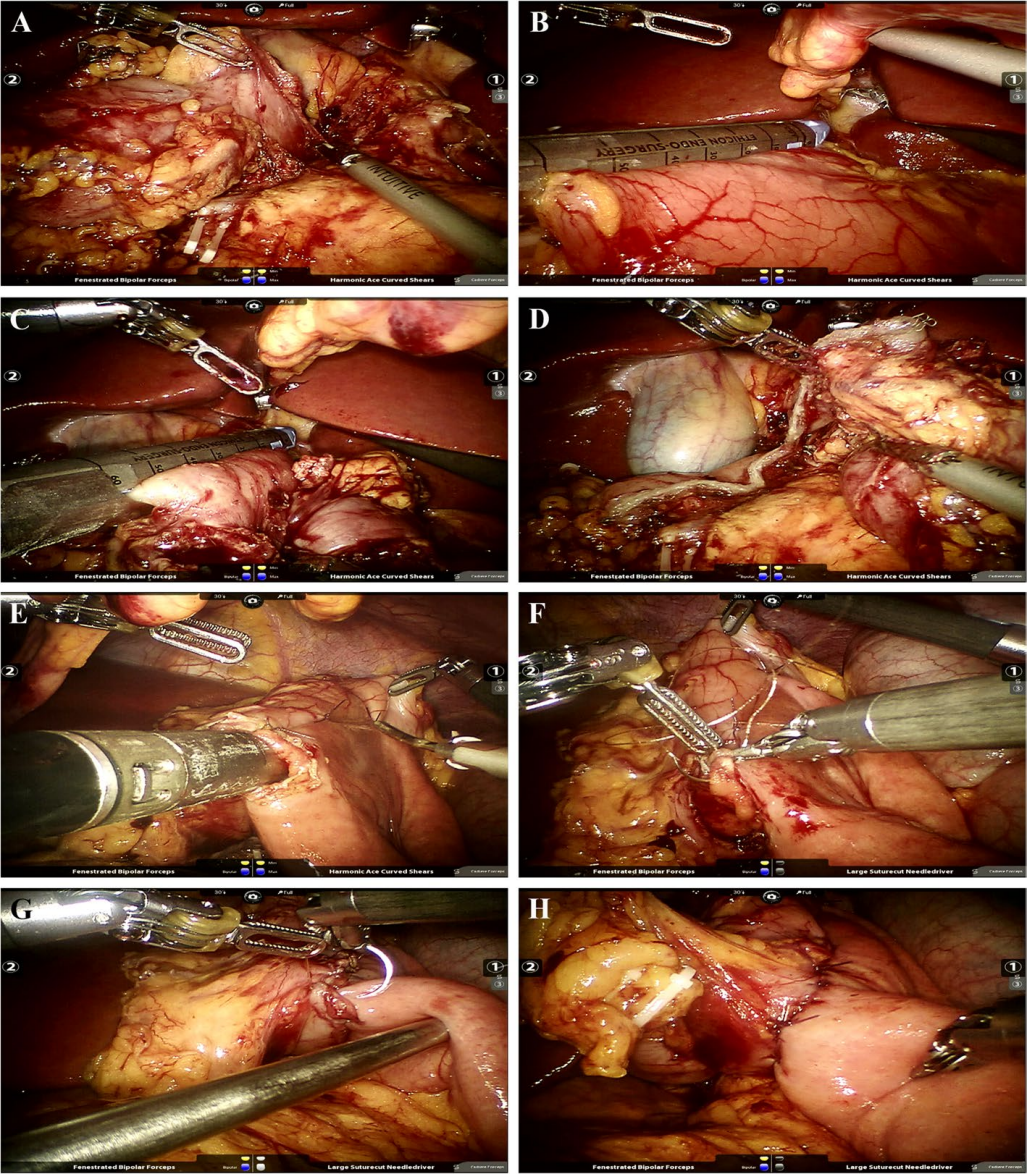
Robotic proximal duodenectomy for a GIST at the first portion of the duodenum. **A**. Dissection of the first portion of the duodenum; **B**. Transection of the stomach; **C**-**D**. Transection of the duodenum; **E**. A side-to-side anastomosis between the stomach and the jejunum; **F**-**G**. Closure of the defect with continuous suture; **H**. View after gastrojejunostomy

**Fig. 3:**
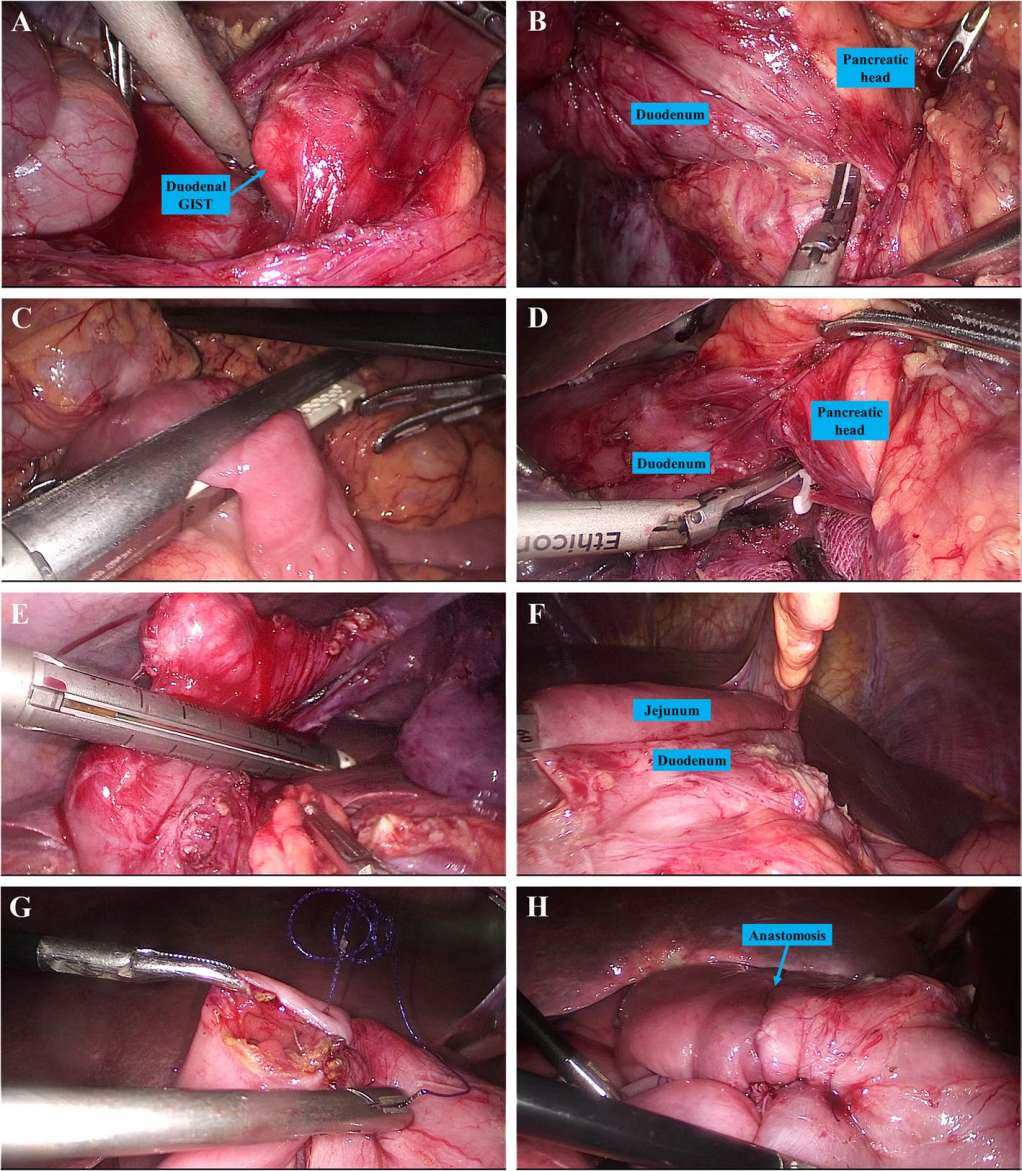
Laparoscopic distal duodenectomy for a GIST at the lower part of the second portion of the duodenum. **A**. Exploration of the duodenal GIST; **B**. Dissection of the duodenum; **C**. Transection of the proximal jejunum; **D**. Separation of the duodenum from the pancreatic head; **E**. Transection of the second portion of the duodenum proximal to the GIST; **F**. A side-to-side anastomosis between the duodenum and the jejunum; **G**. Closure of intestinal defect with continuous suture; **H**. View after duodenojejunostomy

After exploration, the gastrocolic ligament was incised and the hepatic flexure of the colon was mobilized downward to fully expose the duodenum. A kocherization was performed to mobilize the duodenum. Wedge resection was performed for the tumor located at the anti-mesenteric edge of the duodenum, mostly at the D2 portion of the duodenum (Fig. 1). The primary defect area of the duodenum was closed by suture or staples. For proximal duodenectomy, D1 resection and distal gastrectomy were performed with or without resection of the upper part of D2 (Fig. 2). A side-to-side anastomosis between the stomach and the jejunum was performed. For Distal duodenectomy, D3 and D4 resection was performed with or without resection of the lower part of D2. A side-to-side anastomosis between the D2 and the jejunum was performed (Fig. 3). A frozen section was used to confirm the resection margin and tumor pathology if not done preoperatively. For patients with high risks of an intestinal fistula or delayed gastric emptying (DGE), a nutritional jejunostomy was performed. A nasogastric tube was routinely placed before the operation with appropriately delayed removal to relieve the pressure of digestive juice for those patients with wedge resection or duodenojejunostomy.

### Postoperative management and follow-up

The vital signs and drainage condition of the patients were monitored dynamically. Early ambulation was adopted on the first postoperative day (POD1) benefited from the minimally invasive approach. For patients received jejunostomy, 5%GNS was administered on POD1 for early enteric nutrition and total enteric nutrition was then adopted. An upper gastrointestinal contrast examination and CT scan were routinely arranged for the patients one week after the operation to determine postoperative complications.

The patients were followed up at the outpatient services every 3-6months with contrast-enhanced CT, MRI or endoscopy in the first year after surgery, and at annually thereafter to recognize recurrence or metastasis. The disease-free survival (DFS) and overall survival (OS) were defined as the interval from the operative date to the date of disease relapse (local recurrence or distant metastasis), last follow-up, or death, whichever happened first.

### Statistical analysis

All data had undergone normality testing. Continuous data conforming to a normal distribution were presented as mean±standard deviation. Otherwise, the median with range was utilized. Categorical data were presented as frequencies and proportions. Should the continuous data adhere to a normal distribution, the t-test was adopted; otherwise, Mann-Whitney U test was employed. The Chi-square test or Fisher exact test served the purpose of comparing intergroup discrepancies for categorical data. A probability (*P*) value less than 0.05 was set as statistical significance. The statistical analysis was performed using SPSS software version 22.0 (SPSS Inc., Chicago, IL, USA).

## Results

A total of 33 patients with primary duodenal GIST were enrolled and retrospectively reviewed (Table 1). Twenty-three patients received MI-LR and ten received LPD/RPD. There were 16 male and 17 female patients. The mean age was 57±9.41 years. There were 22 symptomatic patients, 16 with gastrointestinal hemorrhage, and 6 with abdominal pain. Eleven patients with asymptomatic duodenal GIST were incidentally diagnosed. The locations of duodenal GIST were D1 (*n*=5), D2 (*n*=21), D3 (*n*=7), D4 (*n*=0). The median largest tumor diameter of duodenal GIST was 4cm (2 -15cm). According to the NIH classification, 20 patients were classified into the low-risk group, 7 patients were into the intermediate-risk group, and 6 patients into the high-risk group. There were 28 spindle cell differentiation tumors and 5 mixed-type differentiation with spindle and epithelioid cells. CD117 was positive in all the GISTs, while CD34 was positive in 26 patients. The mitotic count was ≤5/50 HPF in 27 tumors and >5/50 HPF in 6 tumors. Patients received MI-LR exhibited less OT (280 vs. 388.5min, *P*=0.004), less EBL (100 vs. 450ml, *P*<0.001), and lower morbidity of postoperative complications (52.2% vs. 100%, *P*=0.013) than LPD/RPD. Compared with patients who underwent MI-LR, patients who underwent LPD/RPD burdened more aggressive tumors with larger size (*P*=0.047), higher classification (*P*<0.001) and more mitotic count/50 HPF(*P*=0.005). All the patients are alive during a median follow-up of 33(1-110) months. No anastomotic stenosis, local recurrence or distant metastasis was observed during the follow-up period in both groups.

**Table 1:** The clinicopathological data of the patients who underwent LPD/RPD and MI-LR for primary duodenal GIST

Variate	Total	LPD/RPD	MI-LR	*P* value
	(*n*=33)	(*n*=10)	(*n*=23)	
Gender				0.465
Male	16(48.5%)	6(60.0%)	10(43.5%)	
Female	17(51.5%)	4(40.0%)	13(56.5%)	
Age	57±9.41	55.7±10.37	57.57±9.15	0.609
Presentation				0.094
GI hemorrhage	16(48.5%)	4(40.0%)	12(52.2%)	
Abdominal pain	6(18.2%)	4(40.0%)	2(8.7%)	
Incidental finding	11(33.3%)	2(20.0%)	9(39.1%)	
Tumor diameter	4(2-15)	5.25(3.5-15)	4(2-8)	0.047
Tumor location				0.101
1^st^ part of duodenum	5(15.2%)	0(0.0%)	5(21.7%)	
2^nd^ part of duodenum	21(63.6%)	9(90.0%)	12(52.2%)	
3^rd^ part of duodenum	7(21.2%)	1(10.0%)	6(26.1%)	
4^th^ part of duodenum	0(0.0%)	0(0.0%)	0(0.0%)	
Histologic type				0.291
Spindle cell	28(84.8%)	10(100.0%)	18(78.3%)	
Mixed type	5(15.2%)	0(0.0%)	5(21.7%)	
Mitotic count/50 HPF				0.005
≤5/50 HPF	27(81.8%)	5(50.0%)	22(95.7%)	
>5/50 HPF	6(18.2%)	5(50.0%)	1(4.3%)	
CD117				-
Positive	33(100.0%)	10(100.0%)	23(100.0%)	
Negative	0(0.0%)	0(0.0%)	0(0.0%)	
CD34				0.646
Positive	26(78.8%)	7(70.0%)	19(82.6%)	
Negative	7(21.2%)	3(30.0%)	4(17.4%)	
Risk NIH classification				<0.001
Very low risk	0(0.0%)	0(0.0%)	0(0.0%)	
Low risk	20(60.6%)	1(10.0%)	19(82.6%)	
Intermediate risk	7(21.2%)	4(40.0%)	3(13.0%)	
High risk	6(18.2%)	5(50.0%)	1(4.3%)	
R-Status				-
R0	33(100.0%)	10(100.0%)	23(100.0%)	
R1	0(0.0%)	0(0.0%)	0(0.0%)	
Tumor rupture				-
No	33(100.0%)	10(100.0%)	23(100.0%)	
Yes	0(0.0%)	0(0.0%)	0(0.0%)	
OT (min)	290(180-584)	388.5(276-584)	280(180-480)	0.004
EBL (mL)	100(20-2300)	450(50-2300)	100(20-1000)	<0.001
Transfusion	11(33.3%)	6(60.0%)	5(21.7%)	0.049
Postoperative complications	22(66.7%)	10(100.0%)	12(52.2%)	0.013
DGE	11(33.3%)	3(30.0%)	8(34.8%)	1
Intestinal fistula	2(6.1%)	0(0.0%)	2(8.7%)	1
Anastomotic leakage	3(9.1%)	3(30.0%)	0(0.0%)	0.022
GI hemorrhage	1(3.0%)	0(0.0%)	1(4.3%)	1
Intra-abdominal infection	12(36.4%)	8(80.0%)	4(17.4%)	0.001
LOS (day)	21(7-88)	28.5(12-88)	19(7-46)	0.094
Long-term outcome				
Local recurrence	0(0.0%)	0(0.0%)	0(0.0%)	-
Distant metastasis	0(0.0%)	0(0.0%)	0(0.0%)	-
Follow-up (month)	33(1-110)	25(1-63)	39(4-110)	0.115

*MI-LR* minimally invasive limited resection, *LPD* laparoscopic pancreaticoduodenectomy, *RPD* robotic pancreaticoduodenectomy, *GIST* gastrointestinal stromal tumor, *GI* gastrointestinal, *HPF* high-power field

All the patients underwent MI-LR with no conversion, including 12 cases of LLR and 11 cases of RLR (Table 2). All of the clinicopathological data of the patients were similar in the LLR and RLR groups, including gender, age, presentation, tumor location, tumor diameter, histologic type, mitotic count/50HPF, CD117, CD34, risk NIH classification. Details of perioperative data and long-term outcome for patients received MI-LR was showed in Table 3. In the LLR group, there were two patients underwent wedge resection with jejunostomy, six proximal duodenectomies with distal gastrectomy and gastrojejunostomy, four distal duodenectomies with duodenojejunostomy and nutritional jejunostomy. In the RLR group, there were six wedge resections with nutritional jejunostomy, one wedge resection with distal gastrectomy and gastrojejunostomy, two proximal duodenectomy with distal gastrectomy and gastrojejunostomy, two distal duodenectomy with duodenojejunostomy and nutritional jejunostomy. R0 resection was achieved and no tumor rupture occurred during the operation for all the patients.

**Table 2:** The clinicopathological data of the patients who underwent LLR and RLR for primary duodenal GIST

Variate	MI-LR	LLR	RLR	*P* value
	(*n*=23)	(*n*=12)	(*n*=11)	(LLR vs. RLR)
Gender				0.68
Male	10(43.5%)	6(50.0%)	4(36.4%)	
Female	13(56.5%)	6(50.0%)	7(63.6%)	
Age	57.57±9.15	55.5±11.74	59.82±4.71	0.268
Presentation				0.3
GI hemorrhage	12(52.2%)	7(58.3%)	5(45.5%)	
Abdominal pain	2(8.7%)	0(0.0%)	2(18.2%)	
Incidental finding	9(39.1%)	5(41.7%)	4(36.4%)	
Tumor diameter	4(2-8)	4.25(2.5-8)	4(2-7)	0.615
Tumor location				0.152
1^st^ part of duodenum	5(21.7%)	4(33.3%)	1(9.1%)	
2^nd^ part of duodenum	12(52.2%)	4(33.3%)	8(72.7%)	
3^rd^ part of duodenum	6(26.1%)	4(33.3%)	2(18.2%)	
4^th^ part of duodenum	0(0.0%)	0(0.0%)	0(0.0%)	
Histologic type				0.317
Spindle cell	18(78.3%)	8(66.7%)	10(90.9%)	
Mixed type	5(21.7%)	4(33.3%)	1(9.1%)	
Mitotic count/50 HPF				1
≤5/50 HPF	22(95.7%)	11(91.7%)	11(100.0%)	
>5/50 HPF	1(4.3%)	1(8.3%)	0(0.0%)	
CD117				-
Positive	23(100.0%)	12(100.0%)	11(100.0%)	
Negative	0(0.0%)	0(0.0%)	0(0.0%)	
CD34				0.317
Positive	19(82.6%)	11(91.7%)	8(72.7%)	
Negative	4(17.4%)	1(8.3%)	3(27.3%)	
Risk NIH classification				0.51
Very low risk	0(0.0%)	0(0.0%)	0(0.0%)	
Low risk	19(82.6%)	10(83.3%)	9(81.8%)	
Intermediate risk	3(13.0%)	1(8.3%)	2(18.2%)	
High risk	1(4.3%)	1(8.3%)	0(0.0%)	

*MI-LR* minimally invasive limited resection, *LLR* laparoscopic limited resection, *RLR* robotic limited resection, *GIST* gastrointestinal stromal tumor, *GI* gastrointestinal, *HPF* high-power field

**Table3:** Perioperative data and long-term outcome of patients with primary duodenal GIST who underwent LLR and RLR

Variate	MI-LR	LLR	RLR	*P* value
	(*n*=23)	(*n*=12)	(*n*=11)	
Procedure				
Wedge resection	9(39.1%)	2(16.7%)	7(63.6%)	0.036
Proximal duodenectomy	8(34.8%)	6(50.0%)	2(18.2%)	0.193
Distal duodenectomy	6(26.1%)	4(33.3%)	2(18.2%)	0.635
Digestive reconstruction				
Gastrojejunostomy	9(39.1%)	6(50.0%)	3(27.3%)	0.4
Duodenojejunostomy	6(26.1%)	4(33.3%)	2(18.2%)	0.64
Nutritional jejunostomy	14(60.9%)	6(50.0%)	8(72.7%)	0.4
R-Status				-
R0	23(100.0%)	12(100.0%)	11(100.0%)	
R1	0(0.0%)	0(0.0%)	0(0.0%)	
Tumor rupture				-
No	23(100.0%)	12(100.0%)	11(100.0%)	
Yes	0(0.0%)	0(0.0%)	0(0.0%)	
OT (min)	280(180-480)	280(210-480)	257(180-450)	0.773
EBL (mL)	100(20-1000)	100(20-1000)	100(20-200)	0.603
Transfusion	5(21.7%)	4(33.3%)	1(9.1%)	0.317
Postoperative complications	12(52.2%)	8(66.7%)	4(36.4%)	0.22
DGE	8(34.8%)	4(33.3%)	4(36.4%)	1
Intestinal fistula	2(8.7%)	2(16.7%)	0(0.0%)	0.478
Anastomotic leakage	0(0.0%)	0(0.0%)	0(0.0%)	-
GI hemorrhage	1(4.3%)	0(0.0%)	1(9.1%)	0.478
Intra-abdominal infection	4(17.4%)	3(25.0%)	1(9.1%)	0.59
LOS (day)	19(7-46)	19.5(7-46)	19(9-38)	0.916
Adjuvant Imatinib	2(8.7%)	2(16.7%)	0(0.0%)	0.478
Long-term outcome				
Local recurrence	0(0.0%)	0(0.0%)	0(0.0%)	-
Distant metastasis	0(0.0%)	0(0.0%)	0(0.0%)	-
Follow-up (month)	39(4-110)	52.5(13-110)	18(4-62)	0.001

*MI-LR* minimally invasive limited resection, *LLR* laparoscopic limited resection, *RLR* robotic limited resection, *GIST* gastrointestinal stromal tumor, *OT* operative time, *EBL* estimated blood loss, *DGE* delayed gastric emptying, *GI* gastrointestinal, *LOS* length of stay

The median OT was 280 (210-480) min and 257 (180-450) min, and the median EBL was 100 (20-1000) mL and 100 (20-200) mL in the LLR and the RLR group separately. The postoperative complications mainly included DGE (LLR 4 cases, 33.3% and RLR 4 cases, 36.4%), intestinal fistula (LLR 2 cases, 16.7%, and RLR 0 case),gastrointestinal hemorrhage (LLR 0 case and RLR 1 case, 9.1%), and intra-abdominal infection (LLR 3 cases, 25.0% and RLR 1 case, 9.1%). The median postoperative length of hospitalization was 19.5(7-46) days in the LLR group and 19(9-38) days in the RLR group. The RLR group had a shorter median follow-up duration (18 vs. 52.5 months, *P*=0.001) because robotic approach has recently become the priority choice for duodenal GIST compared to laparoscopic approach in our center.

## Discussion

Resection is the curative treatment for primary duodenal GISTs with or without adjuvant imatinib (IM), including endoscopic resection (ER) and surgical resection. Compared to surgical resection, ER is less invasive and has a similar OS [11, 12]. Nevertheless, considering the high incidence of the positive resection margin and perforation, ER is recommended for duodenal GIST smaller than 2cm [13]. In the present study, ER was not considered since the smallest diameter of duodenal GISTs was 2cm.

Surgical resection for duodenal GISTs could be divided into extended resection and limited resection [14]. Due to the complex anatomic structures in this area, including the duodenum, the head of pancreas, the duodenal papilla, the common bile duct and the main pancreatic duct, PD is a widely accepted method for duodenal GIST [15]. Though the mortality has been significantly reduced in recent decades, PD is still associated with a high incidence of postoperative complications and decreased long-term quality of life. Therefore, LR including wedge resection, segmental duodenectomy, or pancreas sparing total duodenectomy has been increasingly utilized. Segmental duodenectomy consists of proximal duodenectomy and distal duodenectomy, with the duodenal papilla as the borderline. Compared to PD, LR has a lower incidence of complications [14, 16]. Of note, it is reported that LR exhibited the similar long-term outcomes with PD [5]. Moreover, the biological behaviors of duodenal GIST appears to be relatively indolent with a low incidence of lymph node metastasis (3.7%) and distant metastasis (7.5%) [12]. And the characteristic of expansive growth would not be a barrier when dissecting duodenum from adjacent organs. In this light, LR emerges as a favorable surgical option for GIST located at the D1, D3, D4, and on the lateral wall of D2 [17–20]. However, PD should be firstly considered for tumors located at the medial wall of D2, due to the proximity to the pancreatic head or ampullar of Vater [2, 19, 21].

With the rapid development of minimally invasive techniques, skillful general surgeons have increasingly performed MI-LR, including LLR and RLR. However, there is limited literature regarding duodenal GIST. Despite the feasibility and safety of MI-LR has been demonstrated by several case series or case reports, the long-term oncological outcomes deserve further evaluated. MI-LR exhibited decreased OT and POS compared with open LR in a retrospective study enrolled 53 patients with D2 or D3 duodenal GIST [22]. Zhou et al. [23] reported a case series of RLR for 17 patients with duodenal GIST with acceptable outcomes. Whereas most of the other studies were case reports or with a small sample size [7, 24]. Besides, the long-term oncological outcomes remain unclear. Some surgeons even concerned that LLR may elevate the risk of anastomotic leakage, stenosis or tumor rupture which subsequently impact on oncological outcomes [25]. Therefore, laparoscopic approach is limited to GIST smaller than 2cm [8]. In the present study, MI-LR was performed in 23 patients and no tumor rupture, anastomotic leakage, and stenosis were observed which indicated that the risk above could be erased by skillful surgeons. No local recurrence or distant metastasis was observed in all 23 patients which indicated that the long-term oncological outcomes were comparable with LPD/RPD. However, it is noteworthy that the indication of LPD/RPD and MI-LR have not been formally established yet. Optimal minimally invasive surgery strategy for duodenal GIST appears to be individually tailored than non-duodenal GIST. For the oncological feasibility and efficacy, we consider that MI-LR is more suitable for duodenal GIST than LPD/RPD if the tumors do not invade pancreatic head or ampullar of Vater. According to the previous studies, minimally invasive wedge resection was considered suitable for GIST located at any part of the anti-mesenteric border of the duodenum with small size [26, 27]. However, we believed that the diameter of tumor base should be the decisive condition for selecting minimally invasive wedge resection or segmental resection instead of tumor size. In other words, the technical challenge of achieving primary closure in the transverse direction would be significantly reduced if the defects in the duodenal wall are smaller than 5cm. Otherwise, minimally invasive segmental duodenectomy should be considered to avoid stenosis of the duodenum. Distal duodenectomy with duodenojejunostomy could be utilized for GIST at infra-papilla and proximal duodenectomy with gastrojejunostomy for super-papilla. In conclude, the indications of MI-LR should be determined based on the diameter of tumor base, tumor site, and the extent of invasion into pancreatic head or ampullar of Vater.

The robotic operative system can theoretically overcome some of the technical limitations of laparoscopy [28]. It provides tridimensional magnified visualization with high resolution, extended range of motion and tremor filtration, which is related to precise dissection and convenient suture. A recent study showed that robotic PD decreased the incidence of major complications for patients with periampullary cancer than open PD [29]. Hirata Y et al. conducted robotic partial duodenectomy for a patient with duodenal GIST and found no complications or readmission within 90 days [30]. Vicente et al. found satisfied oncological outcomes of patients with duodenal GIST received robotic duodenal enucleations. But the sample size was too small to avoid accidental event with only 3 patients [31]. In the present study, seven patients underwent robotic wedge resection with satisfied clinical and oncological outcomes. Tumor rupture has been recognized as a factor that increases the risk of recurrence [32]. The constraints associated with forceps manipulation during laparoscopic resection can occasionally heighten the risk of tumor rupture. Robotic surgery systems present significant advantages in the treatment of duodenal GIST. With the multi-angle joints and flexible, stable grasping capabilities, the risk of tumor rupture can be minimized effectively. In instances of wedge resection, repairing the defects of the duodenal wall typically requires hand-sewn suturing. The intricacy of the duodenal-pancreatic region poses a considerable challenge to achieving primary closure. Robotic surgical systems offer a solution by furnishing unparalleled precision and maneuverability.

DGE is one of the most common postoperative complications in MI-LR (34.8%). Extended dissection of the duodenum during kocherization might be an important factor associated with DGE. A side-to-side gastrojejunostomy was performed for one patient who underwent wedge resection and no DGE was noticed. Gastrojejunostomy might be an alternative method to prevent DGE for wedge resection. In the study, nutritional jejunostomy was routinely performed for distal duodenectomy and wedge resection without gastrojejunostomy for treating DGE. It can provide total enteric nutrition and shorten the recovery course.

Imatinib plays a critical role in treating GISTs [33]. A retrospective study enrolling 1000 patients with GIST indicated that imatinib significantly prolonged the OS compared to the pre-imatinib area [34]. In the National comprehensive cancer network (NCCN) guidelines, imatinib is recommended for patients with intermediate or high risks of recurrence [35]. Therefore, the effective adjuvant TKI therapy greatly supports the MI-LR for duodenal GIST. So, one patient with high risk of recurrence and one with intermediate risk received adjuvant imatinib therapy after MI-LR in the present study.

This retrospective study has some limitations. Firstly, the sample size of the present study was small because of the rare occurrence of duodenal GIST and strict patient selection criteria. Furthermore, the limited number of cases undergoing conventional open LR in our center hindered a meaningful comparison with MI-LR. Therefore, a multiple-center trial that recruits more patients with duodenal GIST will be necessary in the future to further evaluate the long-term outcomes of MI-LR.

## Conclusion

Minimally invasive limited resection is an optional treatment for primary duodenal GIST with satisfactory short-term and long-term oncological outcomes.

## Data Availability

The datasets used during the current study are available from the corresponding author on reasonable request.

## Abbreviations

GIST: Gastrointestinal stromal tumor
LR: Limited resection
MI-LR: Minimally invasive limited resection
LLR: Laparoscopic limited resection
RLR: Robotic limited resection
PD: Pancreaticoduodenectomy
LPD: Laparoscopic pancreaticoduodenectomy
RPD: Robotic pancreaticoduodenectomy
HPF: High-power field
DGE: Delayed gastric emptying
IM: Imatinib
ER: Endoscopic resection
GI: Gastrointestinal
OT: Operative time
EBL: Estimated blood loss
LOS: Length of stay
EUS: Endoscopic ultrasonography
CT: Contrast-enhanced computed tomography
MRI: Magnetic resonance imaging
NIH: National Institutes of Health
POD1: First postoperative day
DFS: Disease-free survival
OS: Overall survival

## References

[1] Miettinen M, Lasota J (2006). Gastrointestinal stromal tumors: pathology and prognosis at different sites. Semin Diagn Pathol.

[2] Johnston FM, Kneuertz PJ, Cameron JL, Sanford D, Fisher S, Turley R, Groeschl R, Hyder O, Kooby DA, Blazer D, Choti MA, Wolfgang CL, Gamblin TC, Hawkins WG, Maithel SK, Pawlik TM (2012). Presentation and management of gastrointestinal stromal tumors of the duodenum: a multi-institutional analysis. Ann Surg Oncol.

[3] Blay JY, Hindi N, Bollard J, Aguiar S, Angel M, Araya B, Badilla R, Bernabeu D, Campos F, Caro-Sanchez CHS, Carvajal B, Carvajal Montoya A, Casavilca-Zambrano S, Castro-Oliden V, Chacon M, Clara M, Collini P, Correa Genoroso R, Costa FD, Cuellar M, Dei Tos AP, Dominguez Malagon HR, Donati D, Dufresne A, Eriksson M, Farias-Loza M, Fernandez P, Frezza AM, Frisoni T, Garcia-Ortega DY, Gelderblom H, Gouin F, Gomez-Mateo MC, Gronchi A, Haro J, Huanca L, Jimenez N, Karanian M, Kasper B, Lopes David BB, Lopez-Pousa A, Lutter G, Martinez-Said H, Martinez-Tlahuel J, Mello CA, Morales Perez JM, Moura David S, Nascimento AG, Ortiz-Cruz EJ, Palmerini E, Patel S, Pfluger Y, Provenzano S, Righi A, Rodriguez A, Salas R, Santos TTG, Scotlandi K, Soule T, Stacchiotti S, Valverde C, Waisberg F, Zamora Estrada E, Martin-Broto J (2022). SELNET clinical practice guidelines for soft tissue sarcoma and GIST. Cancer Treat Rev.

[4] Casali PG, Blay JY, Abecassis N, Bajpai J, Bauer S, Biagini R, Bielack S, Bonvalot S, Boukovinas I, Bovee J, Boye K, Brodowicz T, Buonadonna A, De Alava E, Dei Tos AP, Del Muro XG, Dufresne A, Eriksson M, Fedenko A, Ferraresi V, Ferrari A, Frezza AM, Gasperoni S, Gelderblom H, Gouin F, Grignani G, Haas R, Hassan AB, Hindi N, Hohenberger P, Joensuu H, Jones RL, Jungels C, Jutte P, Kasper B, Kawai A, Kopeckova K, Krakorova DA, Le Cesne A, Le Grange F, Legius E, Leithner A, Lopez-Pousa A, Martin-Broto J, Merimsky O, Messiou C, Miah AB, Mir O, Montemurro M, Morosi C, Palmerini E, Pantaleo MA, Piana R, Piperno-Neumann S, Reichardt P, Rutkowski P, Safwat AA, Sangalli C, Sbaraglia M, Scheipl S, Schoffski P, Sleijfer S, Strauss D, Strauss SJ, Hall KS, Trama A, Unk M, van de Sande MAJ, van der Graaf WTA, van Houdt WJ, Frebourg T, Gronchi A, Stacchiotti S, Esmo Guidelines Committee E, clinicalguidelines@esmo.org GEa. Gastrointestinal stromal tumours: ESMO-EURACAN-GENTURIS Clinical Practice Guidelines for diagnosis, treatment and follow-up. Ann Oncol 2022;33(1):20-33. 10.1016/j.annonc.2021.09.005. 10.1016/j.annonc.2021.09.005 34560242

[5] Wei YZ, Cai ZB, Zhu CL, Zhou YM, Zhang XF (2021). Impact of Surgical Modalities on Long-term Survival Outcomes of Patients with Duodenal Gastrointestinal Stromal Tumor. Ann Surg Oncol.

[6] Dubois C, Nuytens F, Behal H, Gronnier C, Manceau G, Warlaumont M, Duhamel A, Denost Q, Honore C, Facy O, Tuech JJ, Tiberio G, Brigand C, Bail JP, Salame E, Meunier B, Lefevre JH, Mathonnet M, Idrissi MS, Renaud  F, Piessen  G, Afc, Group FW (2021). Limited Resection Versus Pancreaticoduodenectomy for Duodenal Gastrointestinal Stromal Tumors? Enucleation Interferes in the Debate: A European Multicenter Retrospective Cohort Study. Ann Surg Oncol.

[7] Portale G, Mazzeo A, Fiscon V (2021). Gist of the 4th Portion of the Duodenum: Laparoscopic Resection with Pancreas Preservation. J Gastrointest Surg.

[8] Sakai A, Kinoshita J, Yamaguchi T, Okamoto K, Moriyama H, Nakamura K, Ninomiya I, Inaki N. Robot-assisted distal gastrectomy for duodenal gastrointestinal stromal tumors adhering to the pancreas: a case report. J Surg Case Rep 2023;2023(2):rjad024. 10.1093/jscr/rjad024. 10.1093/jscr/rjad024 PMC9902203 36755930

[9] Fletcher CD, Berman JJ, Corless C, Gorstein F, Lasota J, Longley BJ, Miettinen M, O'Leary TJ, Remotti H, Rubin BP, Shmookler B, Sobin LH, Weiss SW (2002). Diagnosis of gastrointestinal stromal tumors: A consensus approach. Hum Pathol.

[10] Lin R, Lin X, Wu W, Wang C, Lu F, Yang Y, Fang H, Chen Y, Huang H (2022). Robotic parenchymal-sparing pancreatectomy and pancreas-sparing duodenectomy avoid pancreaticoduodenectomy for benign and low-grade malignant tumours. Langenbecks Arch Surg.

[11] Kim GH, Choi KD, Gong CS, Lee IS, Park YS, Han M, Na HK, Ahn JY, Lee JH, Jung KW, Kim DH, Song HJ, Lee GH, Jung HY (2020). Comparison of the treatment outcomes of endoscopic and surgical resection of GI stromal tumors in the stomach: a propensity score-matched case-control study. Gastrointest Endosc.

[12] Yan H, Liu X, Yin L, Han H, Jin Y, Zhu X, Liu Z (2022). Effects of endoscopic therapy and surgical resection on long-term survival outcomes in patients with duodenal gastrointestinal stromal tumors: a surveillance, epidemiology, and end result program analysis. Surg Endosc.

[13] Ye LP, Mao XL, Zheng HH, Zhang Y, Shen LY, Zhou XB, Zhu LH (2017). Safety of endoscopic resection for duodenal subepithelial lesions with wound closure using clips and an endoloop: an analysis of 68 cases. Surg Endosc.

[14] Shen Z, Chen P, Du N, Khadaroo PA, Mao D, Gu L (2019). Pancreaticoduodenectomy versus limited resection for duodenal gastrointestinal stromal tumors: a systematic review and meta-analysis. BMC Surg.

[15] Casali PG, Abecassis N, Aro HT, Bauer S, Biagini R, Bielack S, Bonvalot S, Boukovinas I, Bovee J, Brodowicz T, Broto JM, Buonadonna A, De Alava E, Dei Tos AP, Del Muro XG, Dileo P, Eriksson M, Fedenko A, Ferraresi V, Ferrari A, Ferrari S, Frezza AM, Gasperoni S, Gelderblom H, Gil T, Grignani G, Gronchi A, Haas RL, Hassan B, Hohenberger P, Issels R, Joensuu H, Jones RL, Judson I, Jutte P, Kaal S, Kasper B, Kopeckova K, Krakorova DA, Le Cesne A, Lugowska I, Merimsky O, Montemurro M, Pantaleo MA, Piana R, Picci P, Piperno-Neumann S, Pousa AL, Reichardt P, Robinson MH, Rutkowski P, Safwat AA, Schoffski P, Sleijfer S, Stacchiotti S, Sundby Hall K, Unk M, Van Coevorden F, van der Graaf WTA, Whelan J, Wardelmann E, Zaikova O, Blay JY, Committee EG, Euracan,  (2018). Gastrointestinal stromal tumours: ESMO-EURACAN Clinical Practice Guidelines for diagnosis, treatment and follow-up. Ann Oncol.

[16] Zhang S, Tian Y, Chen Y, Zhang J, Zheng C, Wang C (2019). Clinicopathological Characteristics, Surgical Treatments, and Survival Outcomes of Patients with Duodenal Gastrointestinal Stromal Tumor. Dig Surg.

[17] Chung JC, Chu CW, Cho GS, Shin EJ, Lim CW, Kim HC, Song OP (2010). Management and outcome of gastrointestinal stromal tumors of the duodenum. J Gastrointest Surg.

[18] Wu TJ, Lee LY, Yeh CN, Wu PY, Chao TC, Hwang TL, Jan YY, Chen MF (2006). Surgical treatment and prognostic analysis for gastrointestinal stromal tumors (GISTs) of the small intestine: before the era of imatinib mesylate. BMC Gastroenterol.

[19] Huang Y, Chen G, Lin L, Jin X, Kang M, Zhang Y, Shi D, Chen K, Guo Q, Chen L, Wu D, Huang P, Chen J (2019). Resection of GIST in the duodenum and proximal jejunum: A retrospective analysis of outcomes. Eur J Surg Oncol.

[20] El-Gendi A, El-Gendi S, El-Gendi M (2012). Feasibility and oncological outcomes of limited duodenal resection in patients with primary nonmetastatic duodenal GIST. J Gastrointest Surg.

[21] Colombo C, Ronellenfitsch U, Yuxin Z, Rutkowski P, Miceli R, Bylina E, Hohenberger P, Raut CP, Gronchi A (2012). Clinical, pathological and surgical characteristics of duodenal gastrointestinal stromal tumor and their influence on survival: a multi-center study. Ann Surg Oncol.

[22] Lee SJ, Song KB, Lee YJ, Kim SC, Hwang DW, Lee JH, Shin SH, Kwon JW, Hwang SH, Ma CH, Park GS, Park YJ, Park KM (2019). Clinicopathologic Characteristics and Optimal Surgical Treatment of Duodenal Gastrointestinal Stromal Tumor. J Gastrointest Surg.

[23] Zhou ZP, Tan XL, Zhao ZM, Gao YX, Song YY, Jia YZ, Li CG (2021). Robotic resection of duodenal gastrointestinal stromal tumour: Preliminary experience from a single centre. World J Gastrointest Oncol.

[24] Lu C, Jin W, Mou Y, Shao H, Wu X, Li S, Xu B, Wang Y, Zhu Q, Xia T, Zhou Y (2020). Optimal Laparoscopic Management and Oncological Outcomes of Gastrointestinal Stromal Tumors in Duodenum: Pancreaticoduodenectomy or Pancreas-Sparing Duodenectomy?. Cancer Manag Res.

[25] Vassos N, Perrakis A, Hohenberger W, Croner RS. Surgical approaches and oncological outcomes in the management of duodenal gastrointestinal stromal tumors (GIST). J Clin Med 2021;10(19). 10.3390/jcm10194459. 10.3390/jcm10194459 PMC8509470 34640476

[26] Lim KT (2021). Current surgical management of duodenal gastrointestinal stromal tumors. World J Gastrointest Surg.

[27] Ojima T, Nakamura M, Hayata K, Kitadani J, Katsuda M, Takeuchi A, Tominaga S, Yamaue H (2020). Laparoscopic Limited Resection for Duodenal Gastrointestinal Stromal Tumors. J Gastrointest Surg.

[28] Mungo B, Hammad A, AlMasri S, Dogeas E, Nassour I, Singhi AD, Zeh HJ, Hogg ME, Lee KKW, Zureikat AH, Paniccia A (2022). Pancreaticoduodenectomy for benign and premalignant pancreatic and ampullary disease: is robotic surgery the better approach?. Surg Endosc.

[29] Meyyappan T, Wilson GC, Zeh HJ, Hogg ME, Lee KK, Zureikat AH, Paniccia A (2022). Robotic approach mitigates the effect of major complications on survival after pancreaticoduodenectomy for periampullary cancer. Surg Endosc.

[30] Hirata Y, Scally C, Badgwell BD, Ikoma N (2022). Robotic excision of gastric and duodenal gastrointestinal stromal tumor. Updates Surg.

[31] Vicente E, Quijano Y, Ielpo B, Duran H, Diaz E, Fabra I, Malave L, Ferri V, Ferronetti A, Caruso R (2016). Robot-assisted resection of gastrointestinal stromal tumors (GIST): a single center case series and literature review. Int J Med Robot.

[32] Joensuu H (2008). Risk stratification of patients diagnosed with gastrointestinal stromal tumor. Hum Pathol.

[33] Klug LR, Khosroyani HM, Kent JD, Heinrich MC (2022). New treatment strategies for advanced-stage gastrointestinal stromal tumours. Nat Rev Clin Oncol.

[34] Cavnar MJ, Seier K, Curtin C, Balachandran VP, Coit DG, Yoon SS, Crago AM, Strong VE, Tap WD, Gonen M, Antonescu CR, Brennan MF, Singer S, DeMatteo RP (2021). Outcome of 1000 Patients With Gastrointestinal Stromal Tumor (GIST) Treated by Surgery in the Pre- and Post-imatinib Eras. Ann Surg.

[35] von Mehren M, Kane JM, Bui MM, Choy E, Connelly M, Dry S, Ganjoo KN, George S, Gonzalez RJ, Heslin MJ, Homsi J, Keedy V, Kelly CM, Kim E, Liebner D, McCarter M, McGarry SV, Meyer C, Pappo AS, Parkes AM, Paz IB, Petersen IA, Poppe M, Riedel RF, Rubin B, Schuetze S, Shabason J, Sicklick JK, Spraker MB, Zimel M, Bergman MA, George GV. NCCN Guidelines Insights: Soft Tissue Sarcoma, Version 1.2021. J Natl Compr Canc Netw 2020;18 (12):1604-1612. 10.6004/jnccn.2020.0058. 10.6004/jnccn.2020.0058 33285515

